# Approval, reimbursement and pricing of high-cost cancer medicines in Australia

**DOI:** 10.1186/2052-3211-8-S1-O9

**Published:** 2015-10-05

**Authors:** Agnes Vitry

**Affiliations:** 1 Quality Use of Medicines and Pharmacy Research Centre, School of Pharmacy and Medical Sciences, Sansom Institute, University of South Australia, Aidelaide, 5001, Australia

## Background

Australian government expenditures on chemotherapy medicines are increasing faster than any other area of health care with an average annual growth rate of 63% from 2009/10 to 2013/14. Funding decisions on new, high-cost cancer medicines are challenging because of insufficient evidence on benefits and risks of new cancer medicines and high prices requested by pharmaceutical companies. We reviewed the current approval, reimbursement and pricing strategies for cancer medicines and the development of new regulatory and funding pathways in Australia.

## Methods

A review of government documents from websites of the Therapeutic Goods Administration (TGA) and the Pharmaceutical Benefits Scheme in Australia, the Food and Drug Administration (FDA) in the USA, the European Medicines Agency (EMA) and National Institute for Health and Care Excellence (NICE) in Europe.

## Results

Of all cancer medicines approved by at least one of three regulatory agencies, the FDA, the TGA and the EMA, between 2010 and 2013, Australia authorised the fewest number of indications for cancer medicines (n=54, 59%) compared to the UK (n= 72, 78%) and the US (n=68, 74%). Australia approved a higher number of indications for funding (n=21, 39%) than NICE in the UK (n=14, 19%). Delays in approval and funding are multifactorial and partly explained by time required for price negotiations. In May 2015, there were special pricing arrangements in place for 23 new cancer medicines including all tyrosine kinase inhibitors and monoclonal antibodies. Since 2010, four cancer medicines were approved for funding via a managed entry scheme requiring submission of more conclusive evidence of cost-effectiveness.

## Conclusions

Australia has implemented rigorous methodologies for assessing the value-pricing of new medicines while developing new managed entry pathways. The confidential nature of the agreements between the Australian government and pharmaceutical companies limits the evaluation of the outcomes with regards to pricing of cancer medicines compared with other countries.

